# BOOSTing patient mobility and function on a general medical unit by enhancing interprofessional care

**DOI:** 10.1038/s41598-021-83444-1

**Published:** 2021-02-22

**Authors:** A. M. Johnson, J. Kuperstein, R. Hogg Graham, P. Talari, A. Kelly, E. E. Dupont-Versteegden

**Affiliations:** 1 Department of Rehabilitation Science, College of Health Sciences, University of Kentucky, 900 S. Limestone Street, Lexington, KY 40536 USA; 2 Department of Health and Clinical Sciences, College of Health Sciences, University of Kentucky, Lexington, USA; 3 Division of Hospital Medicine, University of Kentucky HealthCare, Lexington, USA; 4 Department of Medicine, Center for Health Services Research, University of Kentucky, Lexington, USA

**Keywords:** Health care, Medical research

## Abstract

Low mobility during hospitalization remains prevalent despite associated negative consequences. The goal of this quality improvement (QI) project was to increase patient mobility and function by adding a physical therapist (PT) to an existing interprofessional care team. A mobility technician assisted treatment group patients with mobility during hospitalization based on physical therapist recommendations. Change in functional status and highest level of mobility achieved by treatment group patients was measured from admission to discharge. Observed hospital length of stay (LOS), LOS index, and 30-day all cause hospital readmission comparisons between treatment group and a comparison group on the same unit, and between cross-sectional comparison groups one year prior were used for Difference in Difference analysis. Bivariate comparisons between the treatment and a cross-sectional comparison group from one year prior showed a statistically significant change in LOS Index. No other bivariate comparisons were statistically significant. Difference in Difference methods showed no statistically significant change in observed LOS, LOS Index, or 30-day readmission. Patients in the treatment group had statistically significant improvements in functional status and highest level of mobility achieved. Physical function and mobility improved for patients who participated in mobility sessions. Mobility technicians may contribute to improved care quality and patient safety in the hospital.

## Introduction

Immobility and inactivity for adult general medicine patients during hospitalization is consistently observed^1^ and remains a dangerously persistent care practice increasing the risk of negative outcomes, especially for patients 65 and older^2^. Research has confirmed that low mobility is a significant independent predictor of adverse outcome in older adults^2^. Inactivity precipitates decline in activities of daily living (ADLs), new institutionalization, or death^1,2^ and this functional decline begins the second day of hospitalization in patients ≥ 70 years old^3^. Similarly, hospitalization is associated with increased risk for developing new or worsening disability regardless of physical frailty^4^ and frequently delays full functional recovery or contributes to new functional disability^5,6^. Unwarranted low mobility during hospitalization is inappropriate and harmful care.

Unfortunately, wide-spread clinical changes across hospitals in the United States (US) and internationally that consistently and regularly increase safe patient mobility continue to lag. Attention and awareness focusing on this problem is increasing thanks to events like the global summit #EndPJparalysis sponsored by Health Service 360 in the United Kingdom, and supported by other groups internationally^7^. However, conflict between fall prevention and promotion of mobility remains a substantial barrier to maintaining patient function during hospitalization and patient safety policy changes served as a disincentive to patient mobility in the United States (US)^8^. Unintended consequences of these fall prevention policies and hospital culture targeting patient safety through immobility prompted renewed focus on patient falls resulting in a “national epidemic of immobility” (pg 759) for hospitalized older adults^8^. Other challenges include: clinicians’ beliefs that patients do not desire mobility during hospitalization, staff not viewing mobility care as their responsibility^9^, absence of routine measurement of mobility in hospitalized patients^10^, lack of specific communication on patients’ functional status among their healthcare providers^11^, and the dangerous physical environment patients must navigate to safely move during hospitalization^12,13^. In the face of these challenges and the specific contextual factors that exist at the patient, provider, unit and facility level, new models of care and redesigned clinical practice must be part of the solution to address the persistent low mobility status quo during hospitalization.

Thus, we designed and implemented a QI project for adult general medicine patients to increase mobility and function during hospitalization. The redesigned clinical practice was developed in partnership with an existing interprofessional care program which targets improvements in care transitions for general medicine patients. The clinical team welcomed the addition of a physical therapist and mobility technician to the team despite no evidence of rehabilitation professionals being associated with the program anywhere else in the US. In contrast to other nurse-driven, physical therapist-led, or multidisciplinary studies on early mobility, physical therapist consultation and recommendations for increased patient mobility began at hospital admission and continued from a consistent physical therapist until patient discharge from the hospital. This updated care model also included a mobility technician tasked with assisting patients to achieve higher levels of mobility daily. Our goal was to increase patient mobility and function during hospitalization by adding a PT and mobility technician to the interprofessional team and determine any organizational benefits from the QI project. To evaluate this QI project, we used a quasi-experimental design and chose outcomes associated with both patient and organizational benefits: change in functional status and highest daily level of mobility from admission to discharge for treatment group patients, 30-day same hospital readmission rate, observed hospital length of stay (LOS), and LOS index (a measure of hospital efficiency).

## Methods

### Study overview

This mixed methods QI project was implemented at the University of Kentucky (UK) HealthCare Good Samaritan hospital from August 2, 2016 to February 3, 2017. Qualitative findings from the project were published separately^14^. We compared quantitative outcomes between two internal medicine care teams on a single hospital unit, and performed comparisons using cross-sectional historical data from the same hospital unit teams one year prior. Both teams existed at baseline and provided standard internal medicine care for hospitalized patients on the unit. Patients received usual care on both internal medicine teams during the QI project, which included bedside rounding with an interprofessional care team and specific discharge planning education. The treatment group received mobility sessions with a mobility technician, under the direction of a physical therapist who tracked patient functional status from admission to discharge and initiated conversations about each patient’s functional status and mobility level during rounds. The comparison group received usual care without mobility sessions from the mobility technician or the physical therapist tracking patient function from admission to discharge. The change in functional status from admission to discharge for treatment group patients only was calculated and other outcome measures included length of stay measures and hospital readmissions.

### Setting

Good Samaritan Hospital is a university affiliated community hospital with 180 beds and is part of the larger UK HealthCare system. The project was implemented on a 30 bed unit designated for general-internal medicine regular floor and telemetry patients. Two internal medicine healthcare teams on the unit provided care for approximately 14 patients per day and were identified as Internal Medicine Team 4 (MD4) and Internal Medicine Team 5 (MD5). Both teams utilized the Interprofessional Teamwork Innovation Model (ITIM) when rounding on hospital patients in addition to Project BOOST (Better Outcomes by Optimizing Safe Transitions) discharge planning techniques in clinical care^15^.

### Project BOOST and ITIM

Project BOOST is a care transition and readmission reduction program designed to improve patient outcomes and control the resulting costs, and was used on the unit prior to the QI project^33^. Hallmarks of the program include an emphasis on discharge planning communication among team members with the patient^15^. The Project BOOST healthcare teams at UK HealthCare typically consist of a Hospital Medicine physician, pharmacist, bedside nurse and nurse case manager, and use ITIM patient rounding, a model developed and implemented at UK hospitals to facilitate regular interprofessional communication and discharge planning^16^.

### Mobility treatment

For the treatment group, a physical therapist (PT) and mobility technician (MT) were added to the MD4 healthcare team to promote greater mobility and function during acute hospitalization, while the MD5 team received only standard of care. The PT evaluated functional status of all eligible patients in the treatment group within 48 h of admission using the Activity Measure for Post-Acute Care (AM-PAC) “6 Clicks” Basic Mobility Short Form^17^. AM-PAC is a valid measure of functional status in hospitalized adults with a variety of diagnoses^17^. The measure includes 6 activities from items assessing ability to turn over in bed, sitting down or standing up from a chair, moving from supine in bed to sitting edge of bed, transferring from bed to chair, walking, and ascending 3–5 stairs. The score is based on observation of or clinical judgement on patient’s level of difficulty performing the activity or the level of assistance with the activity on a scale from 1 (unable to do the activity or total assistance required) to 4 (no difficulty or no assistance required from another person). The sum of scores for each item provides a raw score ranging from 6, the lowest functional status requiring total assistance on all items, to 24, the highest functional status where patients are independent with function^18^. The PT continued to track patient functional status throughout hospitalization, entering a daily AM-PAC score immediately before or after ITIM rounding based on chart review, communication with the MT and healthcare team, and/or observation of patient function. This ensured an accurate AM-PAC score was maintained in the electronic health record (EHR) within 48 h of discharge. Any changes in patient functional mobility were communicated during ITIM rounding with the healthcare team and daily mobility goal setting was encouraged by the PT using the Johns Hopkins-Highest Level of Mobility (JH-HLM) scale^19,20^. The JH-HLM uses an ordinal scale that captures mobility milestones starting at 1 which is only lying in bed, the lowest score, and progresses through bed activities, sitting edge of bed, transferring out of bed, standing, taking a few steps, up to 8 which is walking 250 or more feet, the highest possible score (17). The highest level of mobility appropriate for each patient was recommended based on the patient’s personal and recent history, current functional status and physical presentation at time of admission. When appropriate, the PT recommended consultation with rehabilitation staff on the unit (physical and occupational therapy).

A trained rehabilitation technician served as the MT and was responsible for encouraging and assisting with patient participation in mobility sessions at the highest mobility level recommended by the PT according to the JH-HLM scale. Examples of a typical mobility session included the MT supervising or physically assisting the patient walking up and down the unit hallway, assisting patients out of bed to a chair for meals while managing the patients lines and tubes safely, guiding and/or physically assisting patients with repeated range of motion movements to the arms, legs, or trunk in seated or standing positions, or some combination of these activities in a single session. The MT’s goal was to assist each patient in the treatment group with mobility over the course of a daily MT work shift (8 h, five days per week), up to three sessions per day. Daily patient mobility session participation was in addition to any typical PT and OT evaluation or treatment. The MT entered a JH-HLM score each shift, based on the patient’s highest mobility level achieved in the mobility session(s)^19,20^.

### Participants

#### Cross-sectional comparison groups

All patients admitted to the unit from August 2, 2015 to February 3, 2016 were eligible for the cross-sectional historical comparison groups. Patients were grouped based on available hospital team designation and patients were arbitrarily assigned to these teams based on available beds. Both patient groups received usual care for the hospital unit, which included ITIM rounding with the healthcare team and discharge planning education. The cross-sectional historical MD4 and MD5 comparison groups were labeled Group 1 and 2 for clarity (Table 1).

**Table 1:** Time period, design, and patient groups.

Time period	Design	Patient groups	
		MD4	MD5
August 2, 2015–February 3, 2016	Cross-sectional historical comparison	Group 1 (standard care)	Group 2 (standard care)
August 2, 2016–February 3, 2017	Quality improvement project	Group 3* (enhanced care)	Group 4 (standard care)

*Designates treatment group who received enhanced model of care including PT tracking functional status and mobility sessions.

#### QI project groups

All patients admitted to the unit from August 2, 2016 to February 3, 2017 were eligible to participate in this QI project with patients in the treatment group admitted to MD4 and in the comparison group to MD5. Patients were grouped based on available hospital team designation and patients were arbitrarily assigned to these teams based on available beds. The MD4 treatment group which participated in the QI project and MD5 comparison group from the same time period were labeled Group 3 and 4 respectively (Table 1). Both groups received usual acute hospital care and ITIM bedside patient rounding, but only the treatment group (Group 3) had a PT participating in ITIM rounds and mobility sessions with the MT.

None of the treatment group patients declined or objected to the PT tracking their functional status and all patients were made aware of the QI project’s goal to improve quality of care. However, patients were allowed to decline participation in mobility sessions during their hospitalization even as their functional status was consistently tracked. Due to conflicts with medical testing, personal needs, severity of illness, or other reasons, patients occasionally declined participation in specific mobility sessions with the MT. In some instances, patients declined all participation in mobility sessions during their entire hospitalization for personal reasons (e.g. the patient felt they did not need it, insisted they were too sick to participate, etc.).

#### Exclusion criteria

Women who were pregnant, individuals under 18 years old, prisoners, patients with non-standard discharge dispositions (e.g. left against medical advice), and patients hospitalized for less than 48 h were excluded from all data collected for this study. As part of the QI project ramp up and ramp down phases, existing Group 3 patients were not offered mobility sessions in week one of the project. Similarly, during the final week of the project, newly admitted patients in Group 3 were not offered the mobility treatment.

### Ethics approval

Approval for the study was obtained from the University of Kentucky Institutional Review Board (IRB) Office of Research Integrity and the project was determined to be quality improvement thus, informed consent was waived by the University of Kentucky Medical IRB committee. All QI procedures were carried out in accordance with relevant guidelines and regulations. Patients had the right to decline participation in the mobility treatment at any time during their hospitalization.

### Data collection and outcome measures

All data were collected in the EHR, stored in the UK Healthcare Enterprise Data Warehouse, and accessed retrospectively with assistance from the UK Center for Health Services Research. Prospective collection of data was not permitted according to the IRB. The unit of analysis was individual patient encounters, since some patients experienced hospital readmission during the study. In order to identify patients retrospectively in the cross-sectional comparison time period and during the QI project, the patient’s internal medicine team (MD4 and MD5) designation during their hospitalization and any AM-PAC and JH-HLM scores found in the EHR documentation were pulled for the specific date ranges. Changes in functional status during hospitalization as measured by AM-PAC and JH-HLM scores were the primary outcome measures for patients in the treatment group. Secondary outcome measures were: observed hospital LOS, LOS index (LOSi), and 30-day same hospital all-cause readmission. LOSi was selected for its association with hospital efficiency and is calculated by dividing observed hospital LOS by expected LOS as calculated using a risk adjustment method based on comorbidity and severity of illness^21,22^. Hospital readmission was defined using a modification of the Centers for Medicare and Medicaid Services methodology for 30-day, all-cause readmission, due to the exclusion of patients with non-standard discharge dispositions^23^.

### Statistical analyses

To determine the comparability of the QI treatment group and the cross-sectional historical comparison data from one year prior, patient characteristics including age, gender, expected LOS, and primary diagnosis category were examined. Additionally, comorbidity using the Elixhauser Index^24^ and resource use using Diagnosis Related Group (DRG) Weight were examined to as they provide a more robust measure of comorbidity and clinical prognosis than a single primary diagnosis^25^. DRG Weight is used in calculating payment for a specific case based on the DRG assigned, representing average resources required to care for cases in that DRG relative to average resources used to treat cases in all DRGs^26^.

Paired t-tests were used to determine the change in AM-PAC and JH-HLM scores from admission to discharge in the treatment group only. The first and last available score for each patient encounter was used to calculate the change. Bivariate comparisons were performed to detect differences in each secondary outcome between the treatment and comparison group during the QI project. Similarly, using the cross-sectional historical comparison data, we compared each secondary outcome between the treatment group (Group 3) and Group 1 and between the comparison group (Group 4) and Group 2. Unpaired t-tests were used for observed hospital LOS and LOS index, and a Fisher’s exact test for hospital readmission. Difference in Difference (DiD) regression approach was used to estimate the effect of the treatment on hospital LOS, LOSi, and 30-day same hospital readmission. DiD is a quasi-experimental analysis approach that can be used to estimate the impact of a treatment that affects one group and not another by comparing data before and after the exposure^27,28^. We defined treatment patients as those that participated in the QI project (Group 3). Patients that did not receive the treatment during the QI project served as controls (Group 4). In the absence of data on patients included in the project prior to implementation, a cross-section of data on different patients from the unit prior to the project was pulled to use as the before QI project data (Groups 1 and 2). The difference between means of outcome variables between the treatment and comparison group from the cross-sectional data (Groups 1 and 2) and during the QI project (Groups 3 and 4) was measured using a regression model interaction term. A key assumption of the DiD design is that, in the absence of the exposure, average outcomes in the treatment and comparison group follow parallel paths over time, provided the treatment and comparison groups are comparable; however, we only had one data point prior to implementation of the QI project available and could not test for this trend^27,28^. All analyses were performed using SAS, version 9.4 (SAS Institute, Inc., Cary, NC).

## Results

After application of exclusion criteria, there were n = 291 patient encounters in the treatment group (Group 3) and n = 284 in the comparison group (Group 4) during the QI project. In the cross-sectional comparison groups prior to the study period, patient encounters in Group 1 had n = 205 and n = 236 patient encounters in Group 2 (Table 2). There were no statistically significant differences in descriptive statistics (age, gender, DRG Weight, comorbidity, or expected LOS) between the groups at both time periods. The top patient diagnoses observed are provided in Table 2.

**Table 2:** Descriptive statistics for all groups.

Internal medicine team	MD4		MD5	
Group designation	Group 1	Group 3*	Group 2	Group 4
Number of Patient Encounters	205	291	236	284
Percent Male	46.3	51.9	48.7	47.9
Average Patient Age	53.6 ± 17.7	55.2 ± 18.2	51.9 ± 17.0	54.3 ± 18.3
DRG Weight	1.2 ± 0.8	1.3 ± 0.8	1.3 ± 1.1	1.2 ± 0.7
Average Elixhauser Index	4.0 ± 2.1	4.2 ± 2.1	4.2 ± 2.2	4.1 ± 2.2
Average Expected LOS (days)	4.8 ± 2.2	5.0 ± 2.5	5.2 ± 2.7	5.0 ± 2.4
% Congestive Heart Failure	15.6	20.3	17.4	16.9
% Cardiac Arrhythmia	16.6	17.5	15.7	18.7
% Hypertension, uncomplicated	50.7	40.9	50.0	38.0
% Hypertension, complicated	11.7	22.3	13.6	22.9
% Other Neurological Disorders	14.1	18.9	15.3	14.1
% Chronic Pulmonary Disease	31.7	29.6	34.3	33.5
% Diabetes, uncomplicated	21.5	11.3	19.5	7.7
% Diabetes, complicated	13.7	23.4	18.3	23.9
% Renal Failure	15.6	19.2	14.8	20.1
% Liver Disease	13.2	16.8	13.6	13.0
% Weight Loss	11.7	15.1	16.9	17.3
% Obesity	18.0	22.0	19.9	20.4
% Fluid and Electrolyte Disorders	48.3	45.7	41.1	47.2
% Alcohol Abuse	16.6	18.6	16.1	16.5
% Drug Abuse	15.1	15.1	16.5	18.3
% Depression	23.4	26.8	28.9	26.4

Average Patient Age, DRG Weight, Average Elixhauser Index, and Average Expected LOS display.

Mean ± Standard Deviation. Descriptive statistics were not statistically significant between groups.

* Designates treatment group.

### Treatment subgroups identified

**Figure 1:**
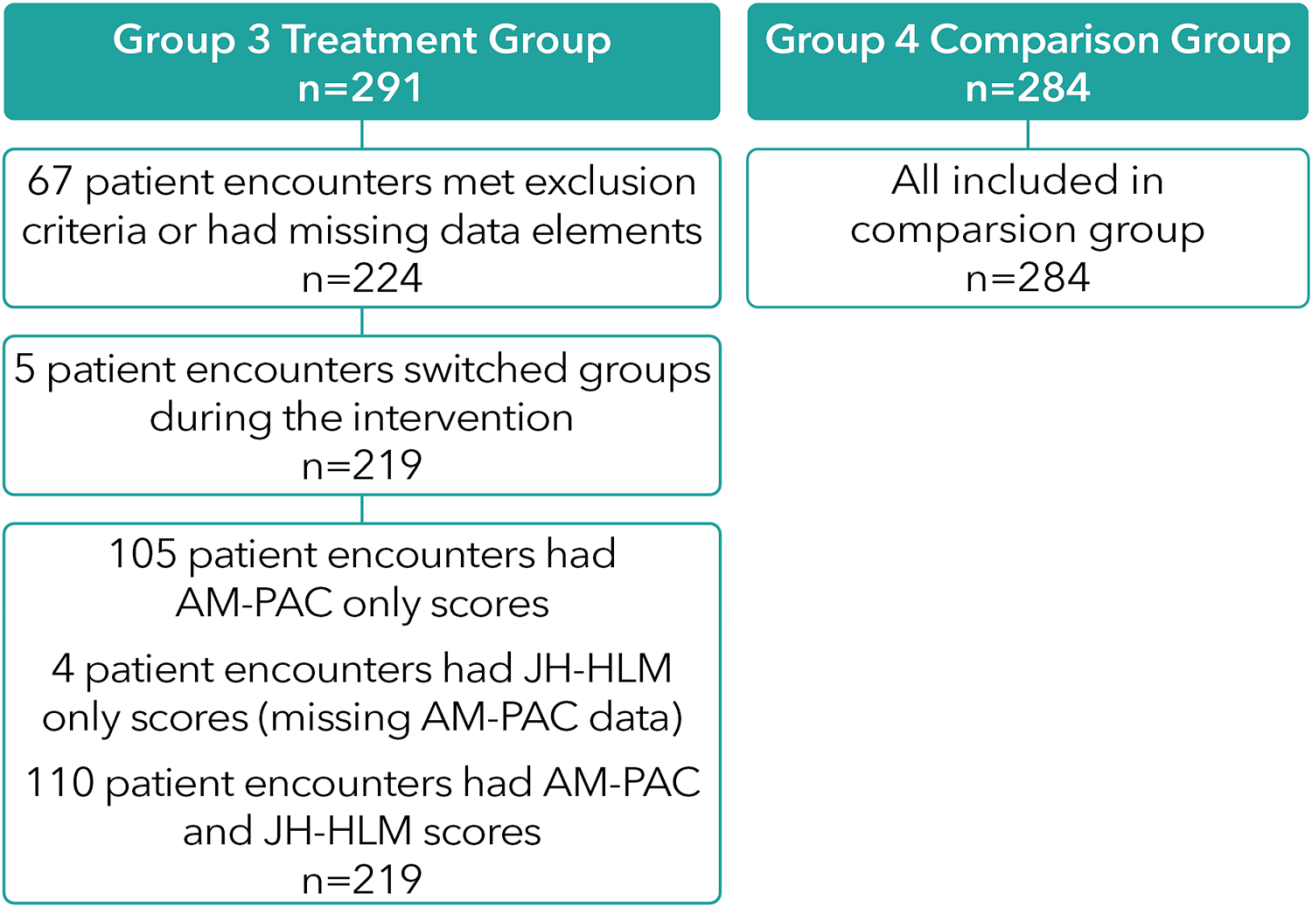
Determining treatment subgroups from the data.

Our results determined two distinct subgroups among patient encounters in the treatment group: those patients who participated in mobility sessions (n = 114) and those who did not (n = 105) (Fig. 1). From the 291 patient encounters in Group 3, some patients were excluded due to the ramp up and down phase of the project, and only 224 had AM-PAC and/or JH-HLM scores in the EHR. Of the 224 patient encounters in Group 3, 105 had only AM-PAC scores and lacked JH-HLM scores, suggesting these patients did not participate in mobility sessions. Five patient encounters were excluded from the 224 patient encounters, as these patients switched internal medicine teams during hospitalization, resulting in n = 219. The final treatment group assumed to have received the full treatment was n = 114 patient encounters (Fig. 1).

### Treatment subgroup comparison within Group 3

Descriptive statistics for patients in the two treatment subgroups found patients were comparable, with the exception of age (Table 3). Patients who participated in mobility sessions were older (*p* < 0.05) (Table 3). Patients who did not participate in mobility sessions had shorter average hospital LOS and shorter LOSi than patients who participated in mobility (Table 3). Results for 30-day same hospital all-cause readmission rates were similar between the treatment subgroups at the conclusion of the study but data did not account for readmissions after the study time frame (Table 3).

**Table. 3:** Treatment subgroup results (Group 3).

	No mobility sessions	Mobility sessions
Number of Patient Encounters	105	114
Age	54.1 ± 15.8	58.6 ± 15.5*
% Male	52.4	49.1
Average Elixhauser	4.5 ± 2.2	4.9 ± 2.2
DRG Weight	1.4 ± 1.1	1.3 ± 0.7
Expected LOS	5.0 ± 2.3	5.4 ± 3.0 (n = 113)
Average observed LOS (days)	5.5 ± 3.6*	8.3 ± 7.3
LOS Index	1.2 ± 0.62*	1.6 ± 1.2 (n = 113)
Hospital readmission rate	16.3% (n = 98)	16.5% (n = 97)

*statistically significant at *p* < 0.05.

### Change in Functional status and Highest level of mobility

For all patients in Group 3, functional status measured by AM-PAC scores increased during hospitalization, although the increase was smaller in patients who did not participate in mobility sessions (Table 4). A comparison of change in AM-PAC score between Group 3 patients who did not participate in mobility sessions and Group 3 patients who did participate in mobility sessions was statistically significant (Table 4). For patients in Group 3 who participated in mobility sessions, AM-PAC and JH-HLM scores increased significantly from admission to discharge (Table 4). The highest level of mobility achieved on average in the last mobility session with the MT was 6.58, which corresponds to a walking activity (taking steps while standing in place) on the JH-HLM.

**Table 4:** Change in function from admission to discharge for Group 3 and two treatment subgroups.

AM-PAC scores	Number of patient encounters	Average first (hospital admission) score	Average last (hospital discharge) score	*p* value*
All Group 3 Patients (Total)	219	18.43	20.3	< 0.05
Group 3 Patients—No Mobility Sessions	105	19.0	20.5	< 0.05
Group 3 Patients -Mobility Sessions	110	17.8	20.3	< 0.05
JH-HLM Scores Group 3 Patients -Mobility Sessions	114	5.84	6.58 (n = 113)	< 0.05

*p value represents statistically significant change from first to last score.

Between group t-test comparison for Group 3 – No Mobility Sessions and Group 3 – Mobility Sessions.

was statistically significant at *p* < 0.05.

**Figure 2:**
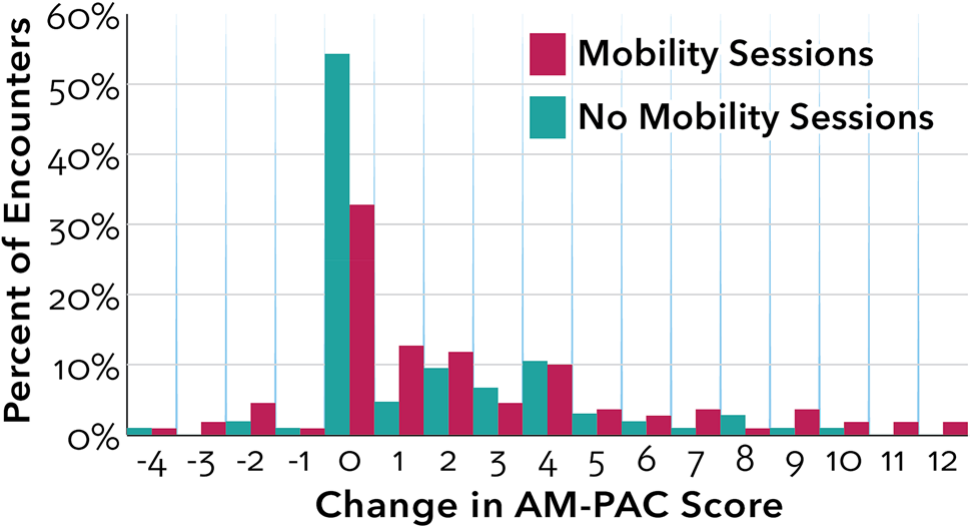
Change in AM-PAC score from admission to discharge, by treatment subgroup.

A histogram effectively displays the change in AM-PAC score for all patients in Group 3 based on their subgroup (Fig. 2). For patients who did not participate in mobility, the majority (67.8%) maintained their admission functional score during hospitalization. In comparison, for patients who participated in mobility sessions, 33.5% of patients maintained their admission functional score, 40.5% of patients increased their score by 1–4 points, and 16.7% of patients increased their score by 5 or more points (Fig. 2).

### Observed LOS, LOS Index, and Hospital readmission rate

The cross-sectional historical comparison data permitted mean observed hospital LOS, LOSi, and 30 day same hospital all-cause readmission rate comparisons between the period before and during the QI project (Table 5). When comparing cross-sectional historical Group 1 data to Group 3 treatment group data, only LOSi results achieved statistically significant reductions. No results were significantly different when comparing cross-sectional historical Group 2 data to Group 4 data, the QI comparison group. Similarly, the DiD estimation did not determine statistically significant differences between treatment and comparison groups for the secondary outcome measures (Table 5). A summary of all results can be found in Table 6.

**Table 5:** Observed LOS, LOSi, hospital readmission comparison and difference in difference results.

	Total	MD4		MD5		Unadjusted DID Interaction Term
		Group 1	Group 3	Group 2	Group 4	
Number of patient encounters	1016	205	291	236	284	
Average observed LOS (days)	6.6 ± 5.7	7.1 ± 6.3	6.2 ± 5.5	6.8 ± 5.4	6.3 ± 5.5	− 0.44
LOSi	1.4 ± 1.0	1.5 ± 1.3	1.3 ± 0.95*	1.3 ± 0.91	1.3 ± 0.93	− 1.49
Hospital readmission rate	16.6%	19.4%	14.6%	16.9%	15.7%	− 0.71

*Denotes statistically significant difference (*p* < 0.05) in LOS Index for comparison between Group 1 and Group.

**Table 6:** Summary of results.

Outcome measure	Statistical analysis	Results	Interpretation
Observed LOS	Unpaired t-test between Groups 1 & 3; Groups 2 &4 Unadjusted DiD (interaction term)	No significant reduction in observed LOS between Groups 1 & 3; DiD interaction term not significant	Observed LOS did not improve for the treatment group or when compared to cross-sectional historical comparison groups across the same time period
LOS Index (LOSi)	Unpaired t-test between Groups 1 & 3; Groups 2 & 4 Unadjusted DiD (interaction term)	Statistically significant reduction in LOSi between Groups 1 & 3; DiD interaction term not significant	LOSi decreased for the treatment group during the QI project but not when compared to improvements determined using the cross-sectional historical comparison groups across the same time period
Hospital Readmission	Fisher’s Exact test between Groups 1 & 3; Groups 2 & 4 Unadjusted DiD (interaction term)	No significant reduction in hospital readmission between Groups 1 & 3; DiD interaction term not significant	Hospital readmission did not improve for the treatment group or when compared to cross-sectional historical comparison groups across the same time period
Change in AM-PAC (Group 3 Patients Only)	Paired t-test	All Group 3 patients improved AM-PAC score from admission to discharge; however, patients who participated in mobility sessions had significantly greater improvement	Treatment group patients who participated in mobility sessions had greater improvement in function from admission to discharge than patients who did not participate in mobility sessions
Change in JH-HLM (Group 3 Patients – Mobility Sessions Only)	Paired t-test	Statistically significant improvement in JH-HLM scores from admission to discharge	Treatment group patients who participated in mobility sessions achieved higher levels of mobility at the end of their hospitalization than at the beginning

## Discussion

This QI project successfully increased patient mobility and function during hospitalization by adding a PT and mobility technician to the interprofessional care team. Although we learned that not all patients participated in mobility sessions, for those patients who did, greater gains in functional status were achieved. This clinically significant prevention of hospital acquired functional decline and improved patient capacity for self-care occurred in patients that experienced a longer hospital LOS compared to patients who did not participate in mobility sessions. The majority of patients not participating in mobility (67.8%) maintained consistent functional status from admission to discharge during hospitalization. One potential explanation for this finding is that those patients had less need for mobility sessions since these patients started at a higher functional level on admission compared to patients who participated in mobility sessions. These results may indicate that patients with greater need for improved mobility and function benefitted from mobility sessions. However, additional research is necessary to explore these findings.

QI projects aimed at increasing mobility led or championed by nursing staff have shown positive results with regard to the prevention of hospital acquired functional decline^20,29,30^. Yet, that strategy relies heavily on a single health profession and underestimates the growing evidence exposing missed or rationed nursing care in acute care hospitals^20,21^. Implementing interventions targeted to already overloaded staff members without removing potential low value care tasks will continue to threaten patient safety and care quality in hospitals^31,32^. For this reason and others, without the PT champion and mobility technician on the unit after the QI project, these efforts were not sustained once the project concluded. Our innovative model leveraging the expertise of a physical therapist in combination with a trained mobility technician generated clinically meaningful change in function, e.g. about 17% of patients showed AM-PAC scores that improved 5 points or greater. The minimally clinically important difference (MCID) in AM-PAC Basic Mobility Short Form, generated from outpatient data in patients with low back pain^33^, ranges from 3.3 to 5.1. Future research is needed to determine if positive changes in functional status and mobility levels are higher with PT-led programs compared to nursing-led programs and to establish the MCID of the AM-PAC Basic Mobility Short Form in acute care patients.

We chose to examine outcomes associated with patient and organizational benefits such as observed hospital LOS, LOS index, and 30-day all cause same hospital readmission in a non-randomized population. Our results indicate the mobility QI project was not responsible for reductions in observed LOS, LOSi, and 30-day all cause hospital readmission, despite evidence that mobility QI projects have had positive results on hospital LOS^20,34^. In a project similar to this study, Wood et al.^35^ showed a longer hospital LOS in patients with a higher average case mix index who received a mobility intervention. In that context, the longer hospital LOS for those patients who received mobility sessions may not be as surprising. Patients who participated in mobility sessions were older, had more comorbidities, and were expected to have longer hospital LOS than those patients who did not participate. In the absence of randomization and tight control for confounding variables in this QI project, patient selection bias by the MT, and self-selection for non-mobilization are possible reasons for our results. LOSi has great potential to surpass observed hospital LOS as a valuable performance metric aligned with health system administrators, rehabilitation research, and QI project champions. However, without the inclusion of patient functional status in expected LOS data used to perform LOSi calculations, it cannot accurately be used as a performance metric. Expected LOS is currently a function of the patient’s DRG and other specific patient characteristics (e.g., typically age, sex, urgency of admission, payer) but would be strengthened significantly by inclusion of a functional measure.

There is a well-established association between patient mobility level and/or functional status and hospital readmission^36–39^. To decrease hospital readmission, interventions that support patient capacity for self-care and improved function are needed. Care transition interventions found to be most successful have multiple program components and include interventions that support patient capacity for self-care^40^. Unfortunately, mobility QI projects and more rigorous studies have not typically evaluated this association between mobility level and/or functional status during index hospitalization and subsequent hospital readmission. While two previous studies have reported reduced readmission rates after implementing QI projects^35,41^, we saw no differences in hospital readmission rates in our study. Larger, more strictly controlled studies focused on hospital readmission and functional status are needed, as well as data-driven QI approaches which more clearly identify patients experiencing hospital readmission and the contributing factors.

This QI project was implemented in a natural clinical environment and it is possible that confounding and other sources of bias influenced these results. For example, confounding may have occurred as patients in the treatment and comparison group were on the same hospital floor during the QI project with physician and nursing staff interchanging frequently. Also, due to physician scheduling policies, eight different hospitalist physicians, including several who were new to the hospital, rotated through the intervention rounding team over the course of the project, potentially leading to a lack of consistency or well-established relationships between physicians and the PT and MT, or to high levels of practice variation that may have impacted LOS results. Indeed, previous research has shown significant variation among hospitalists with respect to hospital LOS and discharge destination^42^. Limited resources prevented the feasibility of tracking physical function from admission to discharge for the comparison group during the QI project. The primary MT experienced an injury during the project and a second MT fulfilled the role for the last two months of the QI project; thus, consistency and availability of the MT became challenging due to rehabilitation department needs. Our readmission data only allowed examination of 30-day same hospital all-cause readmission, eliminating the ability to evaluate patient readmission at other hospital facilities. Finally, data were collected prospectively but accessed retrospectively per IRB restrictions, which may have led to errors in collecting AM-PAC and JH-HLM scores documented in the EHR.

The addition of a physical therapist and mobility technician to an existing interprofessional care team improved patient physical function for patients who participated in mobility sessions. However, organizational benefits such as changes in observed hospital LOS, LOSi, and 30-day hospital readmission were not found. Consistent evaluation of patient function to identify potential hospital acquired functional decline and the addition of novel approaches to maintain or improve patient function in acute care are needed to prevent adverse consequences of immobility. The addition of the PT to the rounding team was not intended to increase skilled PT interventions for patients, but rather was a novel solution to overcome the outdated and persistent low mobility status quo in hospitals. Mobility technicians, under the direction of a physical therapist, have an unrealized role in acute care.
